# Integrative Multi-Omics and Single-Cell Analysis Prioritizes YWHAB Within Ubiquitination-Related Transcriptional Programs in Endometrial Carcinoma

**DOI:** 10.3390/genes17101316

**Published:** 2026-10-21

**Authors:** Jiaxi Wang, Hongwen Zhu, Yue Li, Yunxia Yang, Wenjuan Wang, Wei Fan, Rongfang Zheng, Xianyun Xu, Tiansheng Qin

**Affiliations:** 1 The Second Hospital and Clinical Medical School, Lanzhou University, Lanzhou 730030, China; 2 Department of Gynecology, The Second Hospital and Clinical Medical School, Lanzhou University, Lanzhou 730030, China; 3 Cuiying Biomedical Research Platform, The Second Hospital and Clinical Medical School, Lanzhou University, Lanzhou 730030, China

**Keywords:** endometrial carcinoma, YWHAB, ubiquitination, single-cell RNA sequencing, multi-omics, epithelial plasticity

## Abstract

Background: Ubiquitination-related transcriptional programs may help characterize epithelial heterogeneity in endometrial carcinoma (EC). We aimed to identify candidates associated with these programs and epithelial plasticity. Methods: We integrated a 43,613-cell atlas from five endometrioid EC samples and two normal endometrial samples with bulk transcriptomic, genetic-association and protein-expression data. Five scoring methods defined a consensus expression score, with HIGH and LOW quartiles defined within the Epithelial + EMT_like lineage. Differential expression and high-dimensional weighted gene co-expression network analysis (hdWGCNA) identified candidates for exploratory Mendelian randomization (MR) and survival-based prioritization among 541 patients from The Cancer Genome Atlas Uterine Corpus Endometrial Carcinoma (TCGA-UCEC) cohort. Repeated nested cross-validation and cross-model feature support were used to evaluate internal performance and candidate stability. Pseudobulk, independently curated ubiquitin–proteasome system (UPS) gene sets and scSurv input masking provided sensitivity analyses. Results: The lineage-restricted screen identified 444 candidates, including 412 detectable in TCGA. GeneCards and curated Reactome and KEGG UCell scores were positively correlated (ρ = 0.519 and 0.579). YWHAB ranked fourth among 412 genes in the top-10% workflow analysis and fifth in the top-20% sensitivity analysis. Among the 12 nominal MR-associated genes, it had the highest machine-learning priority. Its single-nucleotide polymorphism (SNP) MR estimate was nominal (odds ratio (OR) = 1.732, *p* = 0.0488) and did not survive multiple-testing correction. Higher bulk YWHAB expression was associated with poorer overall survival (HR = 2.188, 95% CI 1.559–3.072). Western blotting showed higher YWHAB protein expression in EC than controls (nine versus nine tissues, *p* = 0.0027), consistent with descriptive Human Protein Atlas (HPA) staining. Single-cell analyses associated YWHAB with RNA processing, proteostasis-related expression and epithelial-state heterogeneity. Conclusions: Lineage-restricted discovery and cross-model prioritization identify YWHAB as an exploratory candidate associated with ubiquitination-related transcriptional programs in EC. Protein-expression evidence supports its further investigation, while causal function and clinical prognostic utility require independent validation.

## 1. Introduction

Endometrial carcinoma (EC) is one of the most common malignancies of the female reproductive system and its clinical burden continues to rise worldwide [1,2]. Many patients with early endometrioid tumors do well after surgery, but recurrence, metastasis, treatment resistance and aggressive progression remain common in a clinically important subset [3,4]. Molecular classification systems, including The Cancer Genome Atlas (TCGA) framework, have improved EC stratification beyond conventional histopathology [5,6]. Even so, tumors with similar histological or molecular features can follow different clinical courses. This gap suggests that tumor-cell states and post-transcriptional or post-translational regulation add another layer to EC heterogeneity.

Tumor progression is not determined only by static genomic alterations. Malignant epithelial cells can occupy proliferative, stress-adaptive, metabolically reprogrammed and epithelial-to-mesenchymal transition-like (EMT_like) states [7,8]. These states may coexist within the same tumor and interact with stromal and immune components. Single-cell RNA sequencing (scRNA-seq) makes it possible to resolve this intratumoral complexity and to detect malignant epithelial programs that are obscured in bulk profiles [9]. In EC, single-cell studies have begun to map heterogeneity across epithelial, immune, stromal and endothelial compartments and have suggested that malignant epithelial states and microenvironmental communication jointly shape disease evolution [10,11,12]. How post-translational regulatory programs are distributed across these malignant epithelial states is still unclear.

Ubiquitination is a central post-translational modification that controls protein stability, signal transduction, DNA damage responses, cell-cycle progression, apoptosis, immune signaling and metabolic adaptation [13,14]. Through the ubiquitin–proteasome system (UPS) and related ubiquitin-dependent pathways, cells maintain proteostasis and remodel signaling networks under stress. Dysregulated ubiquitination has been implicated in uncontrolled proliferation, proteotoxic stress tolerance, immune escape and treatment resistance [15,16]. In gynecological malignancies, ubiquitin ligases, deubiquitinases and proteasome-related pathways also affect tumor growth and drug response [17]. These observations make ubiquitination-related transcriptional programs relevant to EC progression.

The biological meaning of a ubiquitination-related transcriptional signal is likely to depend on cellular context [18]. In one epithelial state, a higher transcriptional score may indicate proteostatic buffering that allows cells to tolerate stress. In another state, the same broad transcriptional signal may accompany proliferation, metabolic remodeling or EMT_like plasticity. Bulk transcriptomic studies average signals from malignant epithelial cells, stromal cells, immune cells and transitional states, so they cannot separate these possibilities. Analyses based only on predefined ubiquitination genes can identify differential expression, but they do not show whether those genes sit in disease-relevant regulatory networks. A cell-state-resolved strategy is therefore needed.

A second issue is candidate prioritization. Differential expression is sensitive to cellular composition, whereas co-expression analysis identifies genes embedded in coordinated modules [19]. Genetic association and Mendelian randomization (MR) address disease susceptibility, while survival-based learning examines expression patterns related to outcome after tumor formation. Integrating these distinct perspectives can prioritize candidates for functional follow-up. Their agreement is informative for discovery, provided that association, prediction and mechanism remain separate.

We therefore asked whether ubiquitination-related transcriptional patterns vary within the epithelial lineage and identify candidates associated with epithelial plasticity. We combined lineage-restricted differential expression with co-expression networks, exploratory genetic association and cross-model survival-based prioritization. This design sought candidates supported by several analytical perspectives rather than a fixed clinical prognostic signature.

We built a single-cell atlas of endometrioid carcinoma and normal endometrium and summarized a broad GeneCards-derived expression set with five scoring methods. Differential expression and high-dimensional weighted gene co-expression network analysis (hdWGCNA) were aligned to the Epithelial + EMT_like lineage. Pseudobulk and independently curated UPS sets assessed sample-level direction and score concordance. Repeated nested TCGA cross-validation then supported cross-model gene ranking, with exploratory MR and tissue protein expression guiding follow-up. YWHAB-focused analyses examined projected survival-related states, RNA processing, metabolic expression programs, pseudotime organization and predicted ligand–receptor communication.

The consensus score measures ubiquitination-related gene expression, with biochemical activity remaining outside its scope. The integrated analyses prioritize YWHAB and define testable associations with epithelial-state programs for subsequent functional investigation.

## 2. Materials and Methods

### 2.1. Data Resources and Single-Cell Processing

Public scRNA-seq matrices were obtained from Gene Expression Omnibus (GEO). From GSE173682, we included five primary endometrioid EC samples: 3533EL/GSM5276933, 3571DL/GSM5276934, 36186L/GSM5276935, 36639L/GSM5276936 and 366C5L/GSM5276937. Metastatic and non-endometrial tumors in that series were excluded. From GSE299623, we included only two normal endometrial samples, C2_10/GSM9042138 and C2_11/GSM9042139, and excluded the six carcinosarcomas. The controls correspond to N1 and N2, aged 49 and 50 years, and are documented as proliferative endometrium. Seven biological sample labels were used; distinct donor identities were not assumed where public metadata were incomplete. Accessions, tissue information, input barcode counts and final atlas counts are provided in Supplementary Table S11.

Single-cell data were processed with Seurat v5.4.0 and Harmony v2.0.3. Cells had more than 500 detected genes, with sample-specific upper limits of 4000–5000 genes and 15,000–25,000 UMIs. Mitochondrial-transcript limits ranged from 3% to 20%, hemoglobin-transcript fractions were below 1%, and predicted doublets were removed. Exact thresholds and per-sample QC counts are listed in Supplementary Table S11. All single-cell analyses used the same post-QC atlas. Following normalization and highly variable gene selection, Harmony used group.by.vars = c(“orig.ident”, “tissue_type”) and theta = c(2, 0). This reduced sample-associated variation in the embedding without imposing a diversity penalty on tissue status or changing the RNA expression matrix. Cell-cycle scores were calculated without regression, and canonical markers and transcriptional signatures supported cell annotation.

### 2.2. Ubiquitination Scoring and Epithelial-Lineage Network Analysis

A GeneCards search for “Ubiquitination” with Relevance Score > 50 yielded 921 unique genes, including 893 represented in the RNA assay (Supplementary Table S2). We used this broad discovery set to summarize ubiquitination-related expression. Whole-atlas expression and scoring analyses provided descriptive tissue and cell-type context. For candidate discovery, the five scores were calculated independently within all 9879 Epithelial + EMT_like cells, including tumor and normal epithelial-lineage cells. RNA expression was LogNormalized with scale.factor = 10,000. AddModuleScore used the RNA feature pool, nbin = 24, ctrl = 100 and seed = 1. AUCell, UCell, singscore and single-sample gene-set enrichment analysis (ssGSEA) were calculated through irGSEA (version 3.3.4) with seed = 123, min.cells = 3, min.feature = 0, maxGSSize = 1000 and kcdf = “Gaussian”. Each component was z-standardized within the lineage, and their equally weighted mean defined the consensus score.

The lineage-specific 25th and 75th percentiles defined Q1/LOW and Q4/HIGH, while the middle 50% was labeled Middle. Only the two extreme quartiles entered differential expression. Seurat FindMarkers used the RNA assay, a two-sided Wilcoxon rank-sum test, min.pct = 0.10 and logfc.threshold = 0. Expression filtering determined the tested genes; Seurat’s Bonferroni correction used all 33,721 RNA features. Candidate upregulation required adjusted *p* < 0.05 and avg_log2FC > 1. Downregulation required adjusted *p* < 0.05 and avg_log2FC < −1. Cell-level tests described exploratory expression patterns rather than independent patient-level replication.

For sample-aware sensitivity analysis, raw counts were summed by sample and Q1/Q4 group. Samples with at least 20 cells in each group entered a paired edgeR (version 4.8.2) analysis. After filterByExpr, trimmed mean of M values (TMM) normalization and robust quasi-likelihood fitting used design ~ sample + group. Gene-level *p* values were Benjamini–Hochberg (BH)-adjusted across the full tested family. Pseudobulk analysis assessed expression direction and sample-level evidence for the 444 single-cell candidates, complementing cell-level discovery (Supplementary Figure S12; Supplementary Table S12).

Score concordance was assessed with Spearman correlation, including within-sample estimates and equal-sample descriptive summaries. Independently curated sensitivity sets were REACTOME_PROTEIN_UBIQUITINATION and KEGG_UBIQUITIN_MEDIATED_PROTEOLYSIS from Molecular Signatures Database (MSigDB) 2026.1.Hs C2. We compared their UCell scores with the GeneCards UCell component in the same lineage cells. These comparisons assessed concordance between expression signatures derived from different gene sets. Candidate discovery used the GeneCards consensus-defined Q1/Q4 groups. Membership and source records are provided in Supplementary Table S13.

The epithelial-lineage hdWGCNA v0.4.12 network used metacells and a soft-thresholding power of 14. Module scores were compared between the lineage-specific HIGH/LOW groups. Two-sided Wilcoxon tests with exact = FALSE compared all six non-grey modules, with BH adjustment across the six comparisons. Modules with mean HIGH−LOW score difference > 0 and BH q ≤ 0.05 were retained. Core genes required module eigengene-based connectivity (kME) > 0.4 within a retained module. Their intersection with the HIGH-upregulated DEGs defined the single-cell candidates independently of TCGA survival outcomes.

Candidate over-representation analysis(ORA) used Gene Ontology (GO) biological process (BP), cellular component (CC) and molecular function (MF), Kyoto Encyclopedia of Genes and Genomes (KEGG) legacy, Reactome and Disease Ontology annotations. GO used org.Hs.eg.db, and KEGG and Reactome memberships were obtained from MSigDB 2026.1.Hs. The RNA assay feature set supplied the gene universe, with identifier mapping and database annotation defining each effective background. Hypergeometric *p* values were BH-adjusted across all eligible terms within each ontology or collection, including terms with no candidate hits. Full tested families and effective gene counts are reported in Supplementary Table S5.

### 2.3. Genetic and Survival-Based Candidate Prioritization

Two-sample MR evaluated genetically predicted candidate-gene expression in relation to EC susceptibility. Exposure data were Whole_Blood cis-expression quantitative trait locus (cis-eQTL) summary data from the Westlake University compilation of Genotype-Tissue Expression (GTEx) V8. The official GTEx Whole Blood tissue sample size is 670; its tissue-specific ancestry composition was unavailable for the exposure data used here. Candidate gene mappings and source information are provided in Supplementary Table S9. The outcome dataset was IEU OpenGWAS ebi-a-GCST90018838, annotated as European ancestry, with 2188 EC cases and 237,839 controls (total *n* = 240,027).

Exposure associations were screened at *p* < 5 × 10^−5^. Cis-eQTL source data covered the transcription start site ±1 Mb. Linkage disequilibrium (LD) clumping used PLINK (version 1.90b6.10) with a 503-person European 1000 Genomes Phase 3 reference, r^2^ = 0.1, a 500-kb window and clump-p1 = 0.99 after exposure screening. Exposure standard errors were reconstructed from reported effects and *p* values. TwoSampleMR v0.7.8 harmonise_data used action = 2. One retained instrument was analyzed by Wald ratio, and multiple instruments by multiplicative random-effects inverse-variance weighting. F statistics were reported as instrument-strength diagnostics. BH and Bonferroni corrections were applied both to completed primary gene-level tests and to the planned 444-candidate family. The latter retained the full planned test count for adjustment. Full instruments, harmonization outcomes and test results are provided in Supplementary Tables S9 and S14. Single-instrument estimates were treated as exploratory prioritization evidence. The outcome dataset and LD reference were European, but exact ancestry alignment with the whole-blood exposure data could not be confirmed. Colocalization was not performed.

Genomic Data Commons (GDC) The Cancer Genome Atlas Uterine Corpus Endometrial Carcinoma (TCGA-UCEC) STAR RNA-seq files were matched to clinical and overall survival (OS) records by patient ID. We retained one primary tumor sample per patient (sample type 01) and required explicit OS status with positive, finite follow-up time. Four of 545 matched primary-tumor patients lacked valid follow-up. Duplicate gene symbols were averaged on the FPKM-UQ scale before log2(FPKM-UQ + 1) transformation. Of the 444 single-cell candidates, 412 matched this matrix. The candidate list was determined without TCGA outcomes; any subsequent survival-based feature screening occurred within the relevant training data.

Mime (R package Mime1, version 0.0.0.9000) compared 117 candidate workflows using outcome-stratified outer three-fold cross-validation repeated 30 times. Within each outer training set, inner three-fold cross-validation ranked the workflows. Univariable Cox screening at Wald *p* < 0.05 was repeated within each inner training subset and again for the outer training refit. Algorithm selection, preprocessing, feature selection and fitting were confined to training data. Algorithm-specific tuning retained native training-only resampling, including ten-fold tuning where required by penalized Cox, CoxBoost, GBM or SuperPC. These tuning folds were separate from the outer three-fold evaluation and inner three-fold workflow comparison. The selected inner workflow was refitted on the outer training set and evaluated on its untouched outer test fold.

Harrell’s concordance index (C-index) was evaluated within outer test folds. Complete repeats were summarized by pooling concordant-pair credit and comparable-pair denominators across the three folds. We reported the repeat distribution, its mean and standard deviation (SD), and empirical 2.5th–97.5th percentiles as split variability. A constant-risk null benchmark had C-index 0.5 under the same folds. This null comparison did not evaluate added value beyond clinical covariates. Patient flow, fold status, workflow selection and performance records are provided in Supplementary Table S15.

For candidate prioritization, valid workflows were ranked by inner performance within each outer fold. Top-10% workflow support was the primary summary; top-20% support assessed threshold sensitivity, retaining boundary ties. Within each outer training set, support equally weighted identifiable model–inner-fold fits among the top-ranked workflows. The resulting gene-support fractions were then averaged with equal weight across evaluable outer folds. Unobservable selection stages and dense input retention were not treated as specific gene selection. These ranks summarized cross-model priority rather than defining a final fixed prognostic signature (Supplementary Table S6).

YWHAB expression was examined separately by univariable Cox regression and Kaplan–Meier (KM) analysis. The Cox hazard ratio (HR) was expressed per one-unit increase in log2(FPKM-UQ + 1). For descriptive KM curves, the first repeat’s first outer training fold supplied the median expression cutoff. That cutoff was applied unchanged to its corresponding outer test fold and the full cohort. Log-rank tests and patient counts were reported separately for all three datasets (Supplementary Figure S11; Supplementary Table S10). These single-gene displays were distinct from machine-learning performance estimates.

### 2.4. Human Protein Atlas (HPA) Immunohistochemical Assessment

Protein-level expression of YWHAB was examined descriptively using HPA immunohistochemical images from nine EC tissues and six non-malignant endometrial tissues. Public staining provided tissue-level context for the institutional Western blot series.

### 2.5. Western Blotting

Western blotting used nine biologically independent EC tissues and nine control endometrial tissues collected at our institution. Total proteins were extracted using WB/Co-IP tissue/cell lysis buffer containing protease inhibitors (AFGL-100; Affinibody, Wuhan, China). Twenty micrograms of protein per sample were separated by SDS-PAGE and transferred onto membranes. Membranes were blocked for 20 min with Instant Ultra-Low-Background Signal-Enhancing Blocking Buffer (Affinibody). Primary antibodies were mouse monoclonal anti-YWHAB/14-3-3 beta (1:1000; YP-mAb-03655; UpingBio, Hangzhou, China) and rabbit monoclonal anti-gamma-tubulin (1:5000; YM8659; ImmunoWay, Suzhou, China). HRP-conjugated secondary antibodies were goat anti-mouse IgG (H+L; 1:18,000; db10003) and goat anti-rabbit IgG (H+L; 1:18,000; db10002; Diagbio, Hangzhou, China). Signals were recorded with a Tanon 5200 Multi system (Tanon, Shanghai, China), quantified in ImageJ (version 1.54f) and normalized to gamma-tubulin. Groups were compared using a two-sided Welch independent-samples *t*-test. Representative bands from three tissues per group and quantification of all 18 independent tissues are presented in the Section 3. Full measurements and the six uncropped blot files are provided in Supplementary Table S18 and Supplementary Figure S15.

### 2.6. Single-Cell Survival-Related Projection and YWHAB-Focused Analyses

Native scSurv v0.2.1 was trained independently of the Mime candidate workflows. Its inputs were STAR unstranded counts and OS labels from the 541 primary-tumor patients, together with counts from all 9879 epithelial-lineage cells. The matrices shared 32,263 genes. Normal lineage cells supplied expression background only and had no survival labels. Primary displays and correlations were restricted to 3597 tumor-derived cells: 3344 Epithelial and 253 EMT_like cells. The native raw_beta_z output supplied the projection scores. The beta_z output differs by translation rather than sign reversal. The model used latent dimension 20, hidden dimension 100 and one encoder/decoder layer, with Poisson single-cell likelihood and Efron survival likelihood. Training used phase-specific learning rates of 0.01/0.01/0.0001, batch sizes 1000/1000/500, an epoch cap of 300 and patience 10. The projection was an annotation of survival-related transcriptional structure, not an observed cellular survival endpoint.

We compared YWHAB expression, the lineage-specific consensus score and the scSurv projection using Spearman correlations within tumor Epithelial and EMT_like cells. Input masking set the encoder’s YWHAB column to zero and excluded it from expression-likelihood terms, retaining input dimensions and library-size factors. Paired full and masked runs used matched initialization and batch order under seeds 20261007, 20261008 and 20261009; the first seed supplied the primary figure. Within-sample summaries and input sensitivity are provided in Supplementary Figure S14 and Supplementary Table S16. Masking assessed dependence on direct YWHAB input without establishing independence from correlated genes.

YWHAB-centered functional enrichment compared the upper and lower expression quartiles separately within tumor Epithelial and EMT_like cells. These YWHAB-defined groups were distinct from ubiquitination-score Q1/Q4. ORA used upregulated genes, while gene-set enrichment analysis (GSEA) used whole-gene differential-expression rankings. ORA and GSEA results and their adjusted *p* values are provided in Supplementary Table S7. ORA *p* values were BH-adjusted across the tested term families before selecting terms for display.

### 2.7. Trajectory, Perturbation, Metabolic and Communication Analyses

Monocle3 v1.4.27 and CytoTRACE v0.3.3 assessed state organization in the 3597 tumor epithelial-lineage cells. Within each partition, the highest-CytoTRACE cell supplied the computational root without a cell-type selection criterion. The principal display highlighted partition 4 (2266 cells) and three selected graph paths. YWHAB expression states used the median within the displayed partition, with expression detection used if the median was zero. The branch heatmap used the common median of its own input cells. These state definitions were separate from lineage-score quartiles and YWHAB expression quartiles. Root selection oriented the computational graph without determining biological origin or transition direction.

Virtual perturbation used the scTenifoldKnk (version 1.0.3)/scTenifoldNet (version 1.4) high-variable-gene network from tumor lineage counts. Network estimation used ten networks, with at most 500 cells per network. Simulated overexpression multiplied YWHAB-associated row and column edge weights by ten. The response statistic compared squared manifold distances with mean background squared distance. Reported *p* values came from a chi-square tail with one degree of freedom, with BH-adjusted values retained in Supplementary Table S7. The plotted response categories used raw *p* thresholds of 0.01, 0.05 and 0.10. Network fold changes were model response measures rather than measured expression changes.

Primary metabolic analyses scored twelve built-in AUCell programs in the 3597 tumor lineage cells, with 119 recorded gene memberships. These were metabolic transcriptional signatures rather than complete KEGG pathway sets. Tumor cells were divided within each cell type by median YWHAB expression, with detected versus undetected expression used when the median was zero. Cell-level Wilcoxon *p* values were BH-adjusted across twelve programs per cell type. A scoring-background sensitivity analysis computed the same programs in the full 9879-cell lineage, with tumor-cell and within-sample summaries. Sample summaries required at least 20 cells per expression group and were descriptive. Supplementary Table S17 reports the primary tumor-lineage results and full-lineage scoring sensitivity; different scoring backgrounds can yield different score magnitudes.

CellChat v2.2.0.9001 inferred ligand–receptor mRNA communication potential. EMT_like cells were split by YWHAB expression; other cell-type labels were retained. Downsampling to a maximum of 2500 cells per group produced a 17,100-cell analysis with 523 EMT_like_YWHAB_high and 1293 EMT_like_YWHAB_low cells. Network strength, interaction counts and pathway roles were interpreted as model-based mRNA patterns, not measured protein signaling (Supplementary Figure S9; Supplementary Table S7).

## 3. Results

### 3.1. Construction of a Single-Cell Atlas of Endometrial Carcinoma

**Figure 1:**
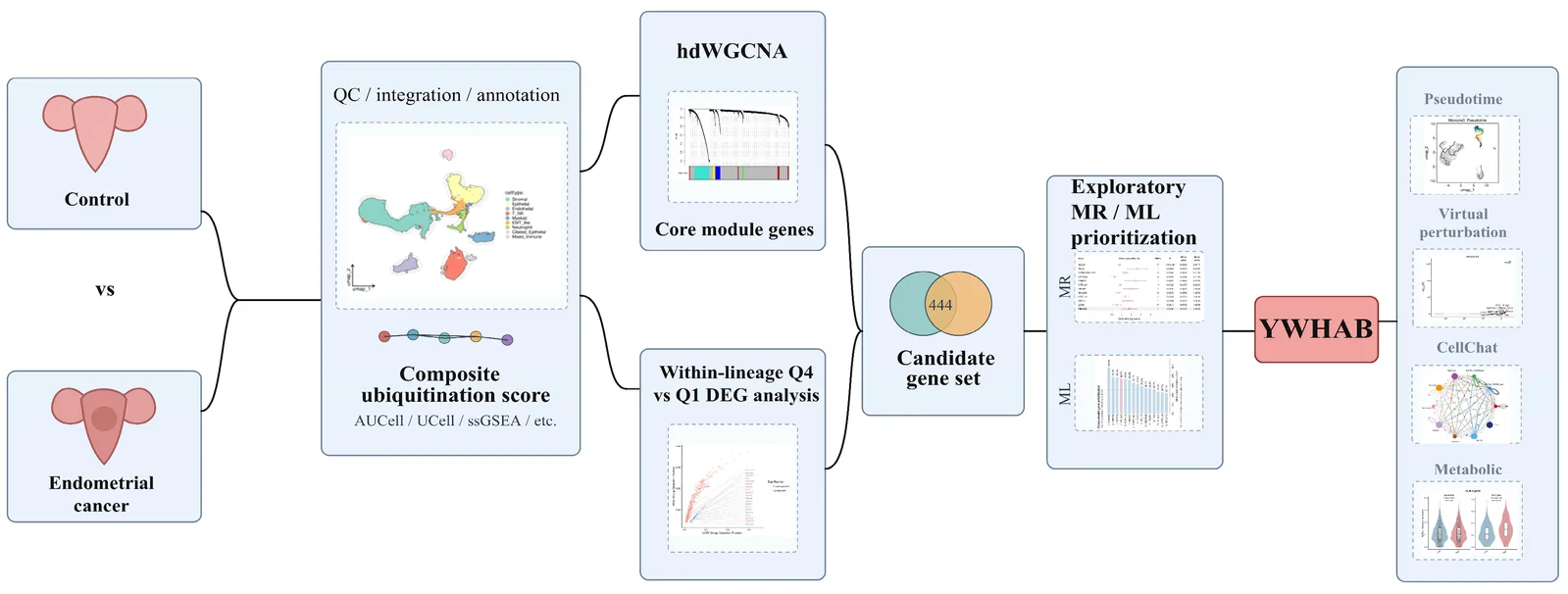
Study workflow. Public single-cell matrices from five endometrioid EC samples and two normal endometrial samples were integrat-ed to construct the atlas. Within the Epithelial + EMT_like lineage, lineage-specific expression scores define Q1/LOW and Q4/HIGH. The 607 HIGH-upregulated DEGs intersect with 1339 genes from five hdWGCNA modules to yield 444 candidates. Expression matching retains 412 genes for TCGA survival-based prioritization, while the 444-gene set enters exploratory MR. Repeated nested cross-validation, cross-model support, MR and tissue protein expression guide YWHAB-focused state analyses. Scores and computational models provide transcriptional and exploratory evidence rather than biochemical or causal validation. Light-blue boxes represent workflow components, whereas the pink box highlights YWHAB as the prioritized candidate. Abbreviations: EC, endometrial carcinoma; EMT_like, epithelial-to-mesenchymal transition-like; DEGs, differentially expressed genes; hdWGCNA, high-dimensional weighted gene co-expression network analysis; TCGA, The Cancer Genome Atlas; MR, Mendelian randomization.

The atlas retained 43,613 cells from 59,822 input barcodes across five primary endometrioid EC samples and two normal proliferative-endometrium samples. Tumor samples contributed 27,527 cells and controls 16,086 cells. Final counts for 3533EL, 3571DL, 36186L, 36639L and 366C5L were 3715, 6317, 4442, 6433 and 6620, respectively. The two controls contributed 7785 and 8301 cells (Supplementary Table S11). The integrative workflow is summarized in Figure 1.

**Figure 2:**
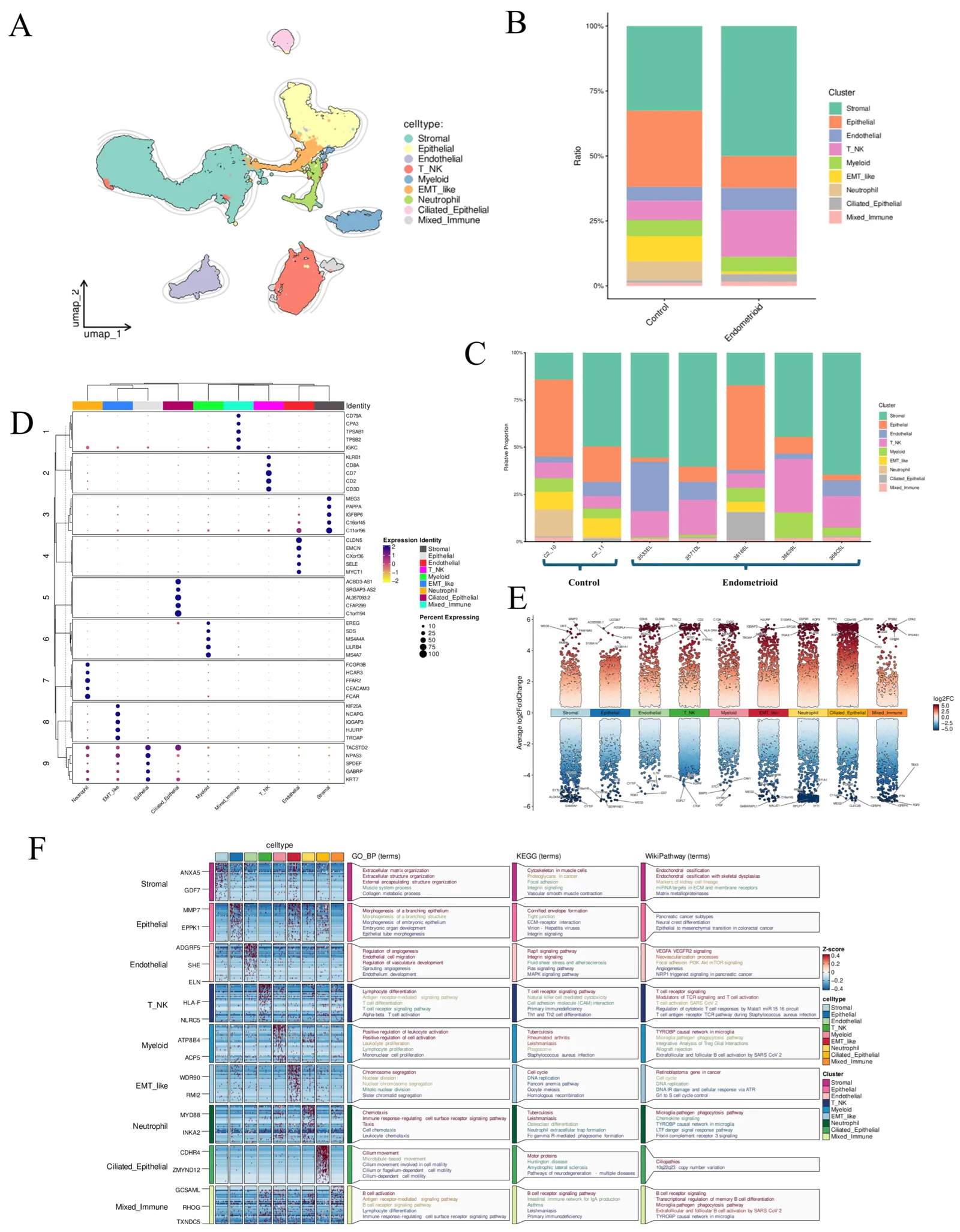
Construction and annotation of the single-cell atlas. (**A**) UMAP of 43,613 retained cells from five endometrioid EC samples and two normal endometrial samples, colored by cell type. (**B**) Cell-type proportions in control and carcinoma tissues. (**C**) Proportions by sample; 3533EL, 3571DL, 36186L, 36639L and 366C5L are tumors from GSE173682, and C2_10 and C2_11 are normal proliferative endometrium from GSE299623. Sample accession, histology and input/post-QC counts are provided in Supplementary Table S11. (**D**) Marker-gene dot plot; size indicates the expressing-cell fraction and color scaled expression. (**E**) Cell-type-enriched marker-gene log2 fold changes. (**F**) Representative transcriptional programs and pathway annotations. Cell-level distributions are descriptive; seven sample labels do not imply 43,613 independent biological replicates. Abbreviations: UMAP, Uniform Manifold Approximation and Projection; EC, endometrial carcinoma; QC, quality control; PCA, principal component analysis; EMT_like, epithelial-to-mesenchymal transition-like; T/NK, T cells and natural killer cells.

Uniform Manifold Approximation and Projection (UMAP) and marker-based annotation resolved nine major populations: stromal, Epithelial, endothelial, T/NK, myeloid, EMT_like, neutrophil, ciliated epithelial and mixed immune cells (Figure 2A–C). Sample-level proportions showed variation in tissue composition. Marker profiles, three-dimensional principal component analysis (PCA) and cell-cycle displays supported the annotations and described proliferative heterogeneity (Figure 2D–F; Supplementary Figures S1–S3; Supplementary Table S1). Epithelial cells expressed MMP7 and EPPK1, whereas EMT_like cells expressed WDR90 and RMI2 in the displayed profiles. Functional annotation associated stromal cells with matrix organization, endothelial cells with angiogenesis and EMT_like cells with chromosome segregation and DNA replication. These displays characterize the observed atlas rather than establish patient-level differences between tumor and normal tissues.

The Epithelial + EMT_like lineage contained 9879 cells: 8063 Epithelial and 1816 EMT_like cells. This lineage included 3597 tumor-derived and 6282 control-derived cells. Its related but distinct transcriptional states provided the common scope for both differential expression and co-expression-based candidate discovery. Tumor-specific projection and functional analyses subsequently used the 3597 tumor-derived lineage cells.

### 3.2. Single-Cell Quantification and Stratification of a Ubiquitination-Related Transcriptional Score

**Figure 3:**
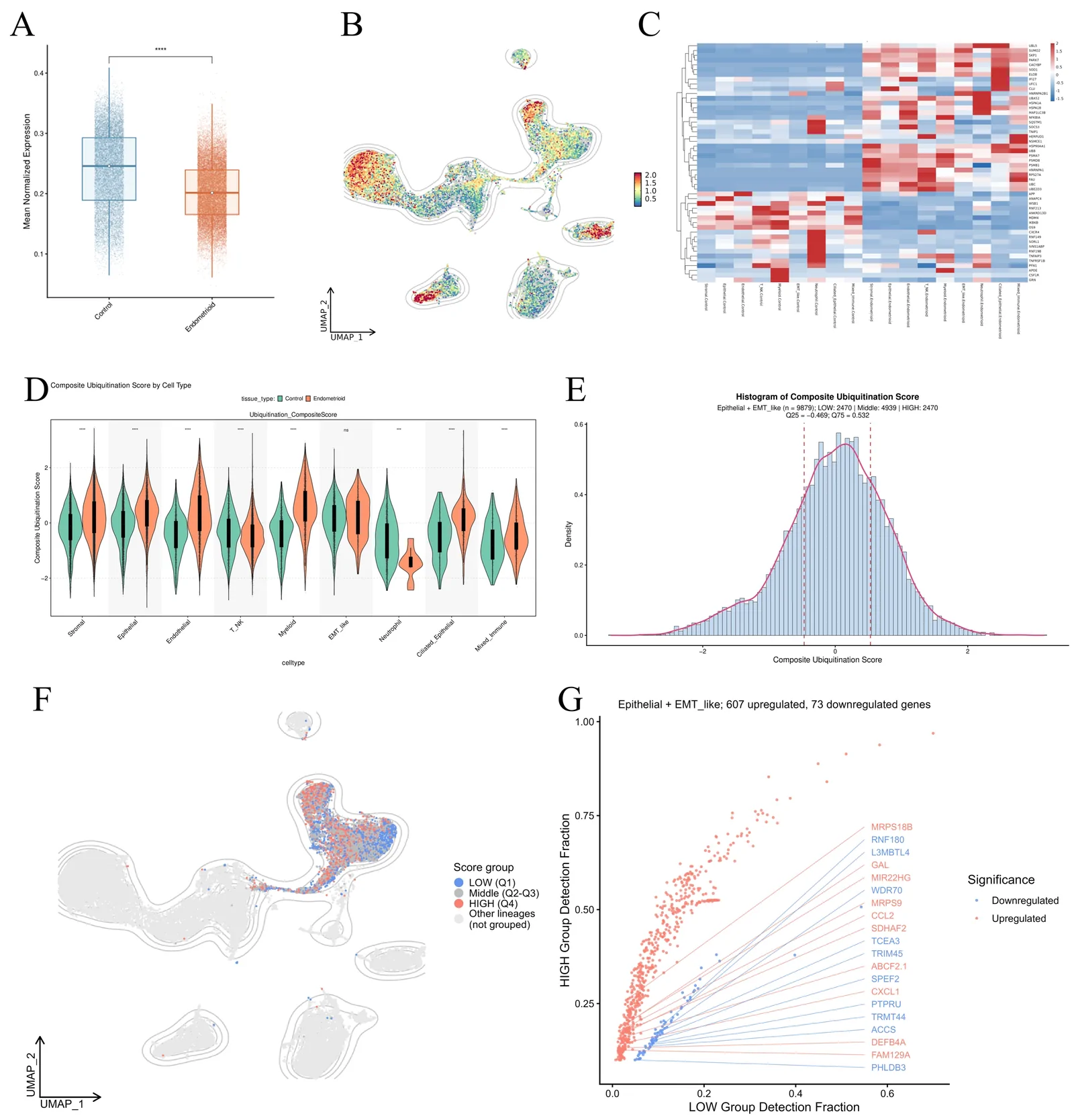
Ubiquitination-related expression patterns and lineage-restricted discovery. (**A**–**D**) Whole-atlas descriptive displays: mean expression of the 893 represented genes, consensus-score UMAP, representative gene expression and tissue comparisons by cell type. Box plots show the median and interquartile range; cell-level annotations describe distributions rather than patient-level replication. *** *p* < 0.001; **** *p* < 0.0001; ns, not significant. (**E**) Lineage-specific consensus scores in 9879 Epithelial + EMT_like cells, with Q1/LOW and Q4/HIGH each containing 2470 cells and Middle containing 4939. The magenta curve represents the smoothed density, and the red dashed lines indicate the 25th and 75th percentile thresholds. (**F**) The lineage-specific groups on the atlas UMAP; other lineages are grey and ungrouped. (**G**) Expressing-cell fractions in LOW and HIGH for significant DEGs with |log2FC| > 1. Color follows average expression fold-change direction, yielding 607 upregulated and 73 downregulated genes. Tests used FindMarkers/Wilcoxon on 9888 genes, with Bonferroni adjustment across 33,721 RNA features. The consensus score represents gene expression rather than biochemical ubiquitination activity. Abbreviations: UMAP, Uniform Manifold Approximation and Projection; EMT_like, epithelial-to-mesenchymal transition-like; DEGs, differentially expressed genes; log2FC, log2 fold change; ns, not significant.

The 893 represented GeneCards genes showed cell-type-dependent expression and scoring patterns across the atlas (Figure 3A–D; Supplementary Figures S4 and S5; Supplementary Table S2). Whole-atlas displays provide tissue and lineage context, while candidate discovery used scores calculated within the 9879-cell epithelial lineage. The lineage-specific consensus score correlated with its five components at ρ = 0.615–0.851 (Supplementary Figure S10; Supplementary Table S8). This agreement describes consistency among expression-scoring methods applied to the same gene set.

Lineage-specific Q1/LOW and Q4/HIGH each contained 2470 cells, with 4939 Middle cells excluded from the extreme-quartile comparison. Score thresholds were −0.46912 and 0.53165 (Figure 3E). Mapping these groups onto the atlas showed their distribution within the epithelial lineage, with other cell types left ungrouped (Figure 3F). Among 9888 tested genes, 607 were HIGH-upregulated and 73 downregulated at Bonferroni-adjusted *p* < 0.05 and |log2FC| > 1 (Figure 3G; Supplementary Table S3).

Paired pseudobulk sensitivity included six samples; 3533EL had only seven Q1 cells and did not meet the 20-cell-per-group requirement. Of 14,091 tested genes, 41 had BH q < 0.05, including eight upregulated and seventeen downregulated genes at |log2FC| > 1 (Supplementary Figure S12). Among the 444 candidates, 342 showed positive direction and twelve reached BH q < 0.05. YWHAB had log2FC = 0.541, nominal *p* = 0.00210 and BH q = 0.1227 (Supplementary Table S12). These results supported directional agreement more strongly than sample-level adjusted significance, so candidate screening was interpreted as exploratory.

Independent UPS sets supported expression-score concordance. Reactome contained 79 members with 63 represented genes, while KEGG contained 135 members with 133 represented genes. Their UCell scores correlated with the GeneCards UCell component at ρ = 0.519 and 0.579, respectively. Both correlations were positive in all seven samples, with ranges of 0.376–0.622 for Reactome and 0.519–0.660 for KEGG (Supplementary Figure S13; Supplementary Table S13). YWHAB was absent from all three scoring sets. These findings support agreement with curated UPS-related expression signatures without validating biochemical ubiquitination activity.

### 3.3. hdWGCNA Identifies Co-Expression Modules Associated with the HIGH Transcriptional-Score State in the Epithelial Lineage

**Figure 4:**
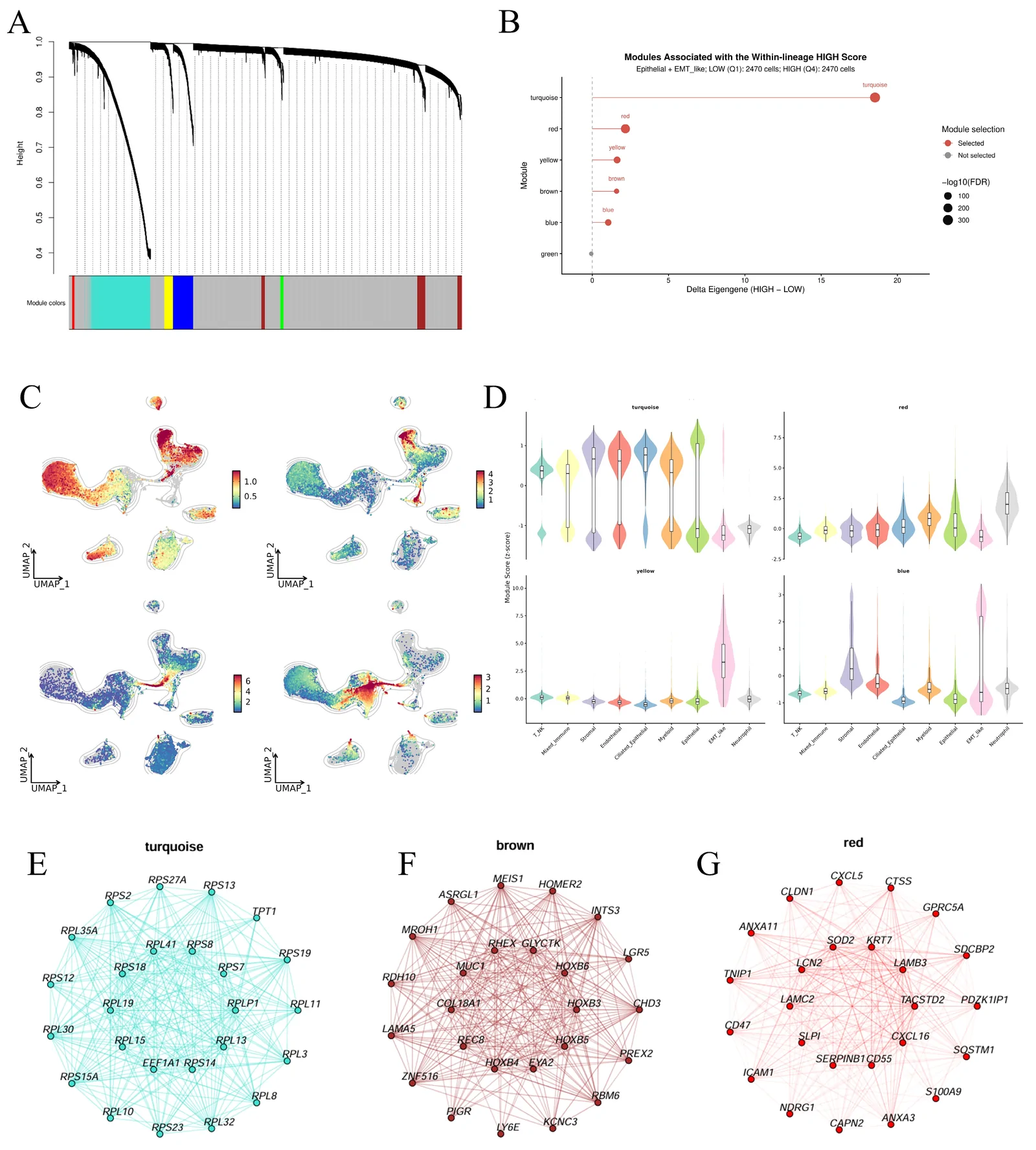
Epithelial-lineage co-expression modules and selection under the lineage-specific HIGH/LOW groups. (**A**) The hdWGCNA dendrogram and module assignments. (**B**) Mean HIGH−LOW differences in module eigengene scores among the lineage-specific quartiles. Two-sided Wilcoxon tests were BH-adjusted across six non-grey modules; point size indicates −log10 q. Positive differences with q ≤ 0.05 selected turquoise, brown, blue, yellow and red; green was excluded. (**C**,**D**) Whole-atlas module projections and cell-type score distributions provide descriptive network context. In (**D**), violin colors distinguish cell types. (**E**–**G**) Representative turquoise, brown and red hub networks. The selected five-module core contains 1339 genes at kME > 0.4 (Supplementary Table S4). Nodes represent genes, and lines indicate co-expression network connections. Abbreviations: hdWGCNA, high-dimensional weighted gene co-expression network analysis; BH, Benjamini–Hochberg; kME, module eigengene-based connectivity.

The epithelial-lineage network resolved six non-grey modules at soft-thresholding power 14 (Figure 4A; Supplementary Figure S6). In the lineage-specific HIGH/LOW comparison, five modules had positive HIGH−LOW differences and BH q ≤ 0.05: turquoise, brown, blue, yellow and red. Green had a negative difference and was excluded from the core candidate set (Figure 4B; Supplementary Table S4). Whole-atlas module projections and cell-type distributions described where the network programs were expressed (Figure 4C,D). These displays provided descriptive context for the lineage-restricted module selection.

The five selected modules contained 1,339 core genes at kME > 0.4: 748 turquoise, 231 brown, 221 blue, 118 yellow and 21 red genes. kME rankings for all six modules describe network membership, with green excluded from candidate discovery (Supplementary Figure S7; Supplementary Table S4). Representative turquoise hubs included RPS27A, RPL41, EEF1A1 and TPT1; brown hubs included MEIS1, LGR5, MUC1 and HOXB-family genes. Red hubs included KRT7, LCN2, SOD2, SQSTM1, CXCL5 and S100A9 (Figure 4E–G). Together, these modules connected the HIGH expression state to translation, epithelial-state organization and stress-associated transcriptional programs.

### 3.4. Intersecting Genes Reveal the Molecular Program of the HIGH Transcriptional-Score State

**Figure 5:**
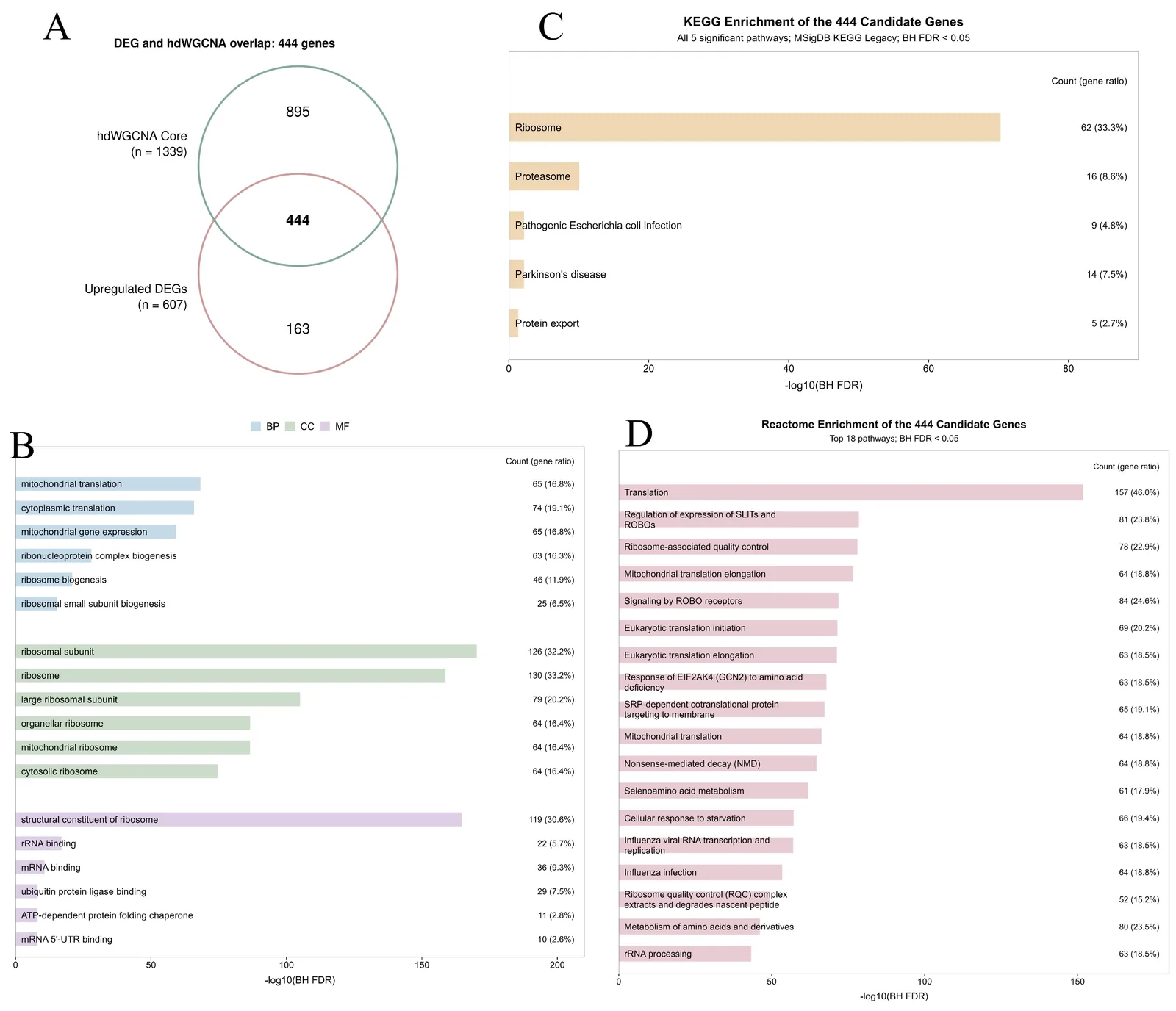
Functional annotation of the 444 lineage-restricted candidates. (**A**) Intersection of 607 HIGH-upregulated DEGs and 1339 core genes from the five selected hdWGCNA modules. The green and red circles represent the hdWGCNA core genes and HIGH-upregulated DEGs, respectively. (**B**) GO over-representation results, with distinct colors for BP, CC and MF. (**C**) KEGG legacy pathway enrichment. (**D**) Reactome enrichment. Horizontal bar lengths show −log10 BH-adjusted *p* values; colors distinguish annotation categories rather than effect direction. Adjustment uses each complete eligible-term family, with effective backgrounds and all tested terms provided in Supplementary Table S5. The 444 candidates include 412 genes detectable in the TCGA machine-learning matrix. Abbreviations: DEGs, differentially expressed genes; hdWGCNA, high-dimensional weighted gene co-expression network analysis; GO, Gene Ontology; BP, biological process; CC, cellular component; MF, molecular function; KEGG, Kyoto Encyclopedia of Genes and Genomes; BH, Benjamini–Hochberg; TCGA, The Cancer Genome Atlas.

Intersecting 607 HIGH-upregulated DEGs with the five-module core of 1339 genes yielded 444 candidates (Figure 5A; Supplementary Table S5). YWHAB had lineage-level log2FC = 1.367 and turquoise-module kME = 0.714. Of the 444 candidates, 412 matched the TCGA expression matrix for survival-based prioritization.

The 444-gene candidate set was enriched for translational and proteostatic functions (Figure 5B–D; Supplementary Table S5). GO included mitochondrial translation, cytoplasmic translation, ribonucleoprotein-complex biogenesis, ribosomal subunits, rRNA binding and ubiquitin-protein ligase binding. KEGG identified ribosome and proteasome pathways. Reactome highlighted translation, ribosome-associated quality control and mitochondrial-translation processes. These annotations link the score-associated network to protein-production and quality-control demands, while reflecting the broad transcriptional scope of the discovery set.

Disease Ontology annotation identified eleven BH-significant terms among 1055 eligible terms, including Diamond–Blackfan anemia and related red-cell aplasia categories (Supplementary Figure S8; Supplementary Table S5). These annotations reflect shared ribosomal gene membership and provide functional context; they do not indicate those diagnoses in the EC samples.

### 3.5. Cross-Model Survival-Based Prioritization and Exploratory MR Identify YWHAB

**Figure 6:**
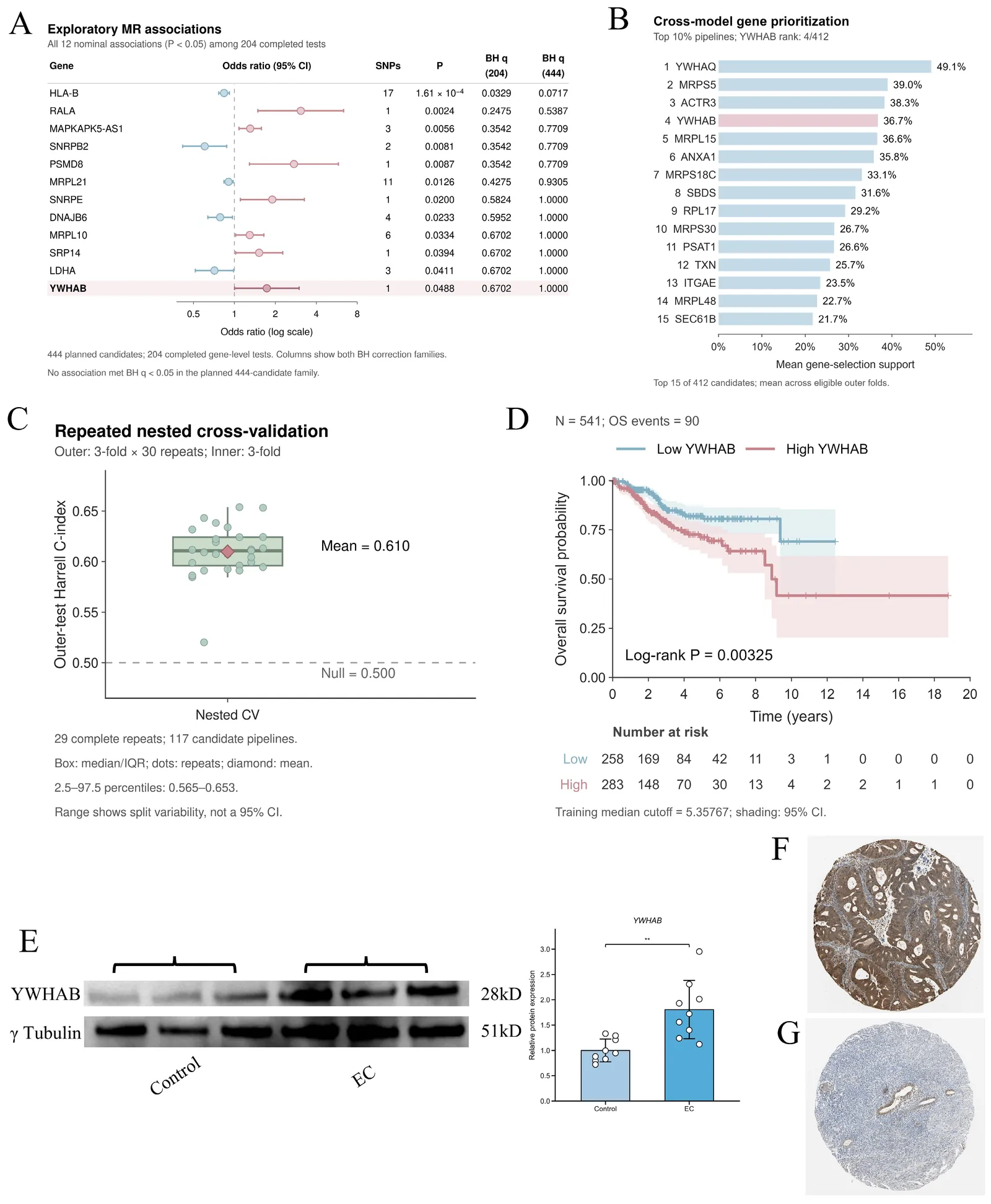
Exploratory prioritization and tissue-level characterization of YWHAB. (**A**) Twelve genes with nominal MR *p* < 0.05, with adjustment across 204 completed tests and 444 planned candidates. YWHAB uses one SNP and does not pass either BH family. Blue and pink points indicate odds ratios below and above 1, respectively; horizontal lines represent 95% confidence intervals, and the vertical dashed line marks an odds ratio of 1. (**B**) Top-10% workflow feature-support ranking; YWHAB ranks 4/412 with mean support 0.367. (**C**) Harrell C-index across 29 complete three-fold repeats, with mean 0.610 and empirical 2.5th–97.5th percentiles 0.565–0.653; these percentiles are not confidence limits. The constant-risk reference is 0.500. Dots represent individual repeats, and the diamond indicates the mean. (**D**) YWHAB-expression KM curves for all 541 patients, including 90 deaths. The cutoff 5.35767 was fixed from the first outer training fold; Low/High groups contain 258/283 patients, and log-rank *p* = 0.00325. Shading indicates 95% survival confidence intervals. Training and corresponding outer-test curves are in Supplementary Figure S11. (**E**) Representative YWHAB and gamma-tubulin bands from three tissues per group, and quantification of all nine EC and nine control tissues. Bars show means and error bars SD; two-sided Welch *p* = 0.0027 (** *p* < 0.01). Uncropped blots are indexed in Supplementary Table S18. (**F**,**G**) Representative HPA staining in tumor and normal endometrium. Neither the nominal MR result nor cross-model priority establishes causal function or a clinically validated signature. Abbreviations: MR, Mendelian randomization; SNP, single-nucleotide polymorphism; BH, Benjamini–Hochberg; C-index, concordance index; KM, Kaplan–Meier; EC, endometrial carcinoma; SD, standard deviation; HPA, Human Protein Atlas.

The 444-candidate MR screen matched 415 genes to the exposure resource, and 204 genes completed primary tests using 568 retained gene–SNP instrument rows. These tests comprised 90 Wald-ratio and 114 inverse-variance-weighted estimates. Twelve genes had nominal *p* < 0.05 (Figure 6A; Supplementary Table S14). HLA-B alone passed BH and Bonferroni correction among the 204 completed tests, while no gene passed either correction across the planned 444-candidate family.

YWHAB retained a single instrument, rs16989474, with F = 77.20. Its Wald-ratio estimate was OR = 1.732 (95% CI 1.003–2.991, *p* = 0.0488), with BH q = 0.6702 for completed tests and q = 1.000 for the planned family. This nominal susceptibility association contributed exploratory priority information rather than corrected genetic evidence. The single instrument precluded conventional multi-instrument heterogeneity, MR-Egger and MR-PRESSO analyses, and colocalization was not performed (Supplementary Tables S9 and S14).

The TCGA-UCEC cohort included 541 primary-tumor patients, 90 OS deaths and 451 censored observations. Reverse Kaplan–Meier median follow-up was 1063 days (2.91 years; 95% CI 973–1120 days). Repeated nested cross-validation evaluated the 412 expression-matched candidates across 117 workflows. Of 90 outer test folds, 89 were evaluable, and 29 repeats completed all three folds. Mean repeat-level Harrell C-index was 0.610 (SD 0.026; median 0.611), compared with 0.500 for the constant-risk null. Empirical 2.5th–97.5th percentiles were 0.565–0.653, describing split variability rather than a confidence interval (Figure 6C; Supplementary Table S15).

Cross-model feature support prioritized YWHAB without relying on one final signature. In the top-10% workflow summary, YWHAB ranked fourth among 412 genes, with mean support 0.367 across 88 folds with identifiable support (Figure 6B; Supplementary Table S6). Top-20% sensitivity placed it fifth, with mean support 0.384 across all 90 folds. The evaluable-fold count is distinct from the number of gene selections. Among the twelve nominal MR-associated genes, YWHAB had the highest machine-learning priority under both summaries. This provided a consistent exploratory basis for selecting it for further characterization.

In the full cohort, higher YWHAB expression was associated with poorer OS in univariable Cox regression (HR per log2-expression unit = 2.188, 95% CI 1.559–3.072, *p* = 5.96 × 10^−6^). Full-cohort KM analysis used the training-fold cutoff of 5.35767 and showed log-rank *p* = 0.00325 (Figure 6D; Supplementary Table S10). The illustrative training fold contained 360 patients and 60 deaths (*p* = 0.0158), and the corresponding outer test fold contained 181 patients and 30 deaths (*p* = 0.0656; Supplementary Figure S11). These single-gene associations were reported separately from cross-validated workflow performance and were not adjusted for clinical covariates.

Western blotting showed higher YWHAB protein expression in EC than in control tissues across nine independent tissues per group. The normalized mean difference was 0.805 (95% CI 0.349–1.261; two-sided Welch *p* = 0.0027). Representative bands show three tissues per group, while quantification includes all eighteen tissues (Figure 6E; Supplementary Table S18). HPA images from nine EC and six non-malignant tissues provided descriptive staining support, with representative images in Figure 6F,G. These protein findings support tissue-level follow-up of the transcriptomically prioritized candidate.

### 3.6. Native scSurv Projection Characterizes Survival-Related Epithelial-State Associations

**Figure 7:**
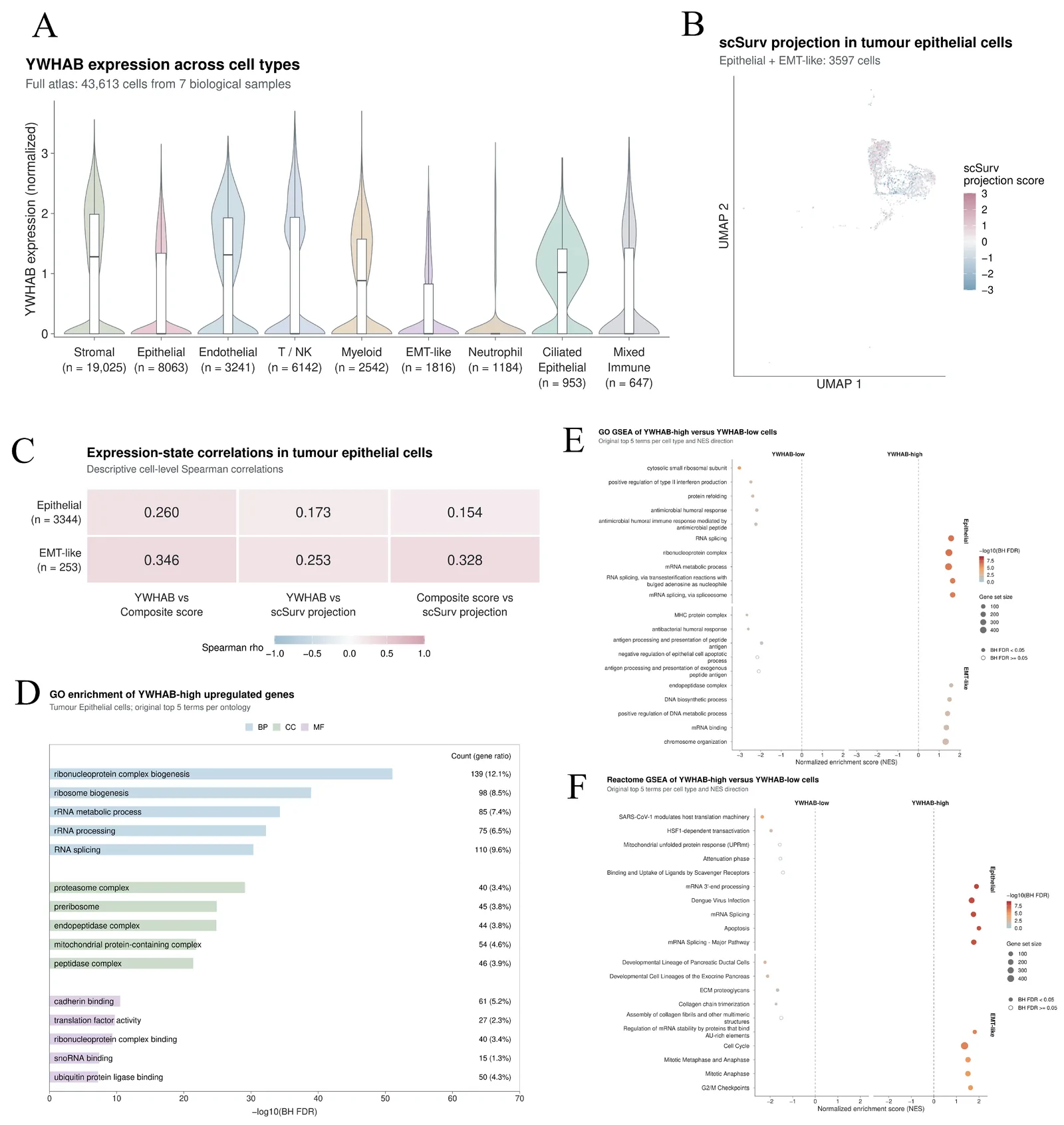
YWHAB expression and associated epithelial-state programs. (**A**) YWHAB expression across all 43,613 atlas cells, grouped by nine annotated cell types. Violins describe cell-level distributions. (**B**) Native raw_beta_z scSurv projection in 3597 tumor-derived lineage cells, trained using 541 patients and 32,263 shared genes with the 9879-cell lineage expression background. (**C**) Spearman correlations among YWHAB expression, the lineage-specific consensus score and the projection in tumor Epithelial (*n* = 3344 cells) and EMT_like (*n* = 253 cells). Coefficients are descriptive cell-level associations. (**D**) GO over-representation of genes upregulated in YWHAB-high Epithelial cells, with separate BP/CC/MF colors and −log10 adjusted *p* bar lengths. (**E**,**F**) GO and Reactome GSEA in YWHAB-defined tumor Epithelial and EMT_like groups. Horizontal position shows normalized enrichment score, color −log10 BH FDR, and point size gene-set size. Functional comparisons use expression quartiles within each cell type: 836 versus 836 Epithelial cells and 64 versus 64 EMT_like cells. These groups differ from ubiquitination-score Q1/Q4. Enrichment results are in Supplementary Table S7; scSurv masking and seed sensitivity are in Supplementary Figure S14 and Supplementary Table S16. Abbreviations: EMT_like, epithelial-to-mesenchymal transition-like; GO, Gene Ontology; BP, biological process; CC, cellular component; MF, molecular function; GSEA, gene-set enrichment analysis; BH, Benjamini–Hochberg; FDR, false discovery rate.

YWHAB expression varied across the nine annotated cell populations (Figure 7A). Independent native scSurv integrated the 541-patient count matrix with the epithelial-lineage expression background and 32,263 shared genes. Its primary tumor display contained 3597 cells, including 3344 Epithelial and 253 EMT_like cells (Figure 7B). This projection described survival-related transcriptional organization without applying the Mime candidate list or a fixed multigene signature to cells.

Within tumor Epithelial and EMT_like cells, YWHAB correlated positively with the lineage-specific consensus score (ρ = 0.260 and 0.346) and the native scSurv projection (ρ = 0.173 and 0.253). Consensus-score correlations with the projection were also positive (ρ = 0.154 and 0.328; Figure 7C; Supplementary Table S7). These cell-level coefficients describe modest within-state associations; sample-level summaries are supplied for sensitivity rather than treating individual cells as independent patients.

Masking YWHAB input before scSurv refitting retained positive YWHAB–projection associations: ρ = 0.116 in Epithelial and 0.243 in EMT_like cells for the primary seed. Full-input and masked results from three seeds are provided in Supplementary Figure S14 and Supplementary Table S16. Direct inclusion of YWHAB creates some dependence between expression and projection; masking addresses that input contribution, while correlated genes can still carry related information. The projection therefore provides exploratory state annotation rather than independent prognostic or mechanistic validation.

### 3.7. YWHAB-Associated Expression Programs Involve RNA Processing and Proteostasis

YWHAB-centered functional comparisons examined its upper and lower expression quartiles within each tumor cell type. Epithelial groups contained 836 cells each, and EMT_like groups 64 cells each. This resolved gene-associated state programs separately from the ubiquitination-score groups used for candidate discovery.

Genes upregulated in YWHAB-high Epithelial cells were enriched for ribonucleoprotein-complex biogenesis, ribosome biogenesis, RNA metabolism and spliceosome-mediated mRNA splicing (Figure 7D). GO GSEA associated the expression-defined states with translation and RNA-processing programs in Epithelial and EMT_like cells (Figure 7E). Reactome GSEA identified mitochondrial-translation, protein-folding and spliceosome-related gene sets (Figure 7F; Supplementary Table S7). These associations place YWHAB within coordinated biosynthetic and proteostatic expression programs and identify processes for functional testing.

The expression-based enrichment and projected-score associations address related but distinct questions. Enrichment characterizes genes co-varying with YWHAB expression, whereas scSurv annotates survival-related transcriptional structure. Their combination localizes candidate biology to epithelial states without assigning direct regulation to YWHAB.

### 3.8. Pseudotime Analysis Reveals a Branched Epithelial-State Continuum

**Figure 8:**
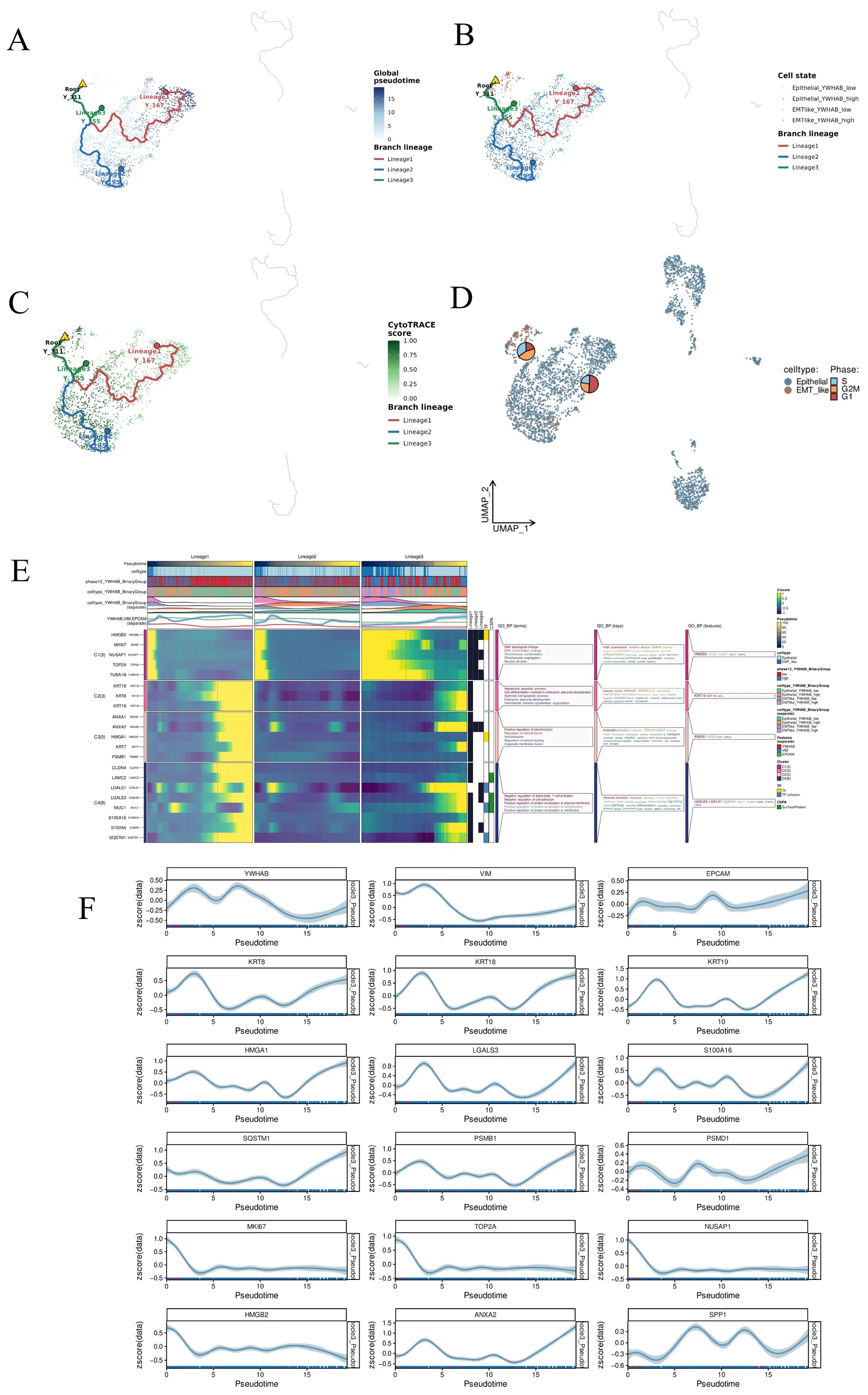
Computational ordering of tumor epithelial-lineage states. (**A**) Monocle3 graph from the 3597-cell tumor lineage, highlighting partition 4 (2266 cells) and three selected paths. Within each partition, the highest-CytoTRACE cell was the computational root; the displayed root is Y_311. The yellow triangle marks the displayed computational root. (**B**) YWHAB expression states defined within the displayed partition by its median, with a detection-based split if the median is zero. (**C**) CytoTRACE distribution. (**D**) Cell-cycle annotations in Epithelial and EMT_like cells. (**E**) Branch-associated expression heatmap and functional annotations, using the common YWHAB median of the heatmap input cells. (**F**) Smoothed representative-gene patterns along the inferred ordering. Graph paths, roots and pseudotime describe computational state organization and do not establish cellular origin or transition direction. Blue curves show smoothed expression trends, with pale shaded bands indicating uncertainty around the fitted trends. Abbreviation: EMT_like, epithelial-to-mesenchymal transition-like.

Monocle3 organized the 3597 tumor Epithelial + EMT_like cells into a branched computational graph. The principal display highlighted partition 4, containing 2266 cells, with three selected paths (Figure 8A–C). Computational roots were selected by the highest CytoTRACE score within each partition, without using cell identity as a criterion. The displayed root in an EMT_like-enriched region therefore reflects score-based orientation rather than a demonstrated biological origin.

YWHAB expression states and CytoTRACE scores varied across the displayed graph. Cell-cycle annotation showed more S-phase and G2/M-phase cells in EMT_like than Epithelial cells (Figure 8D). Branch heatmaps and smoothed gene profiles described variation in YWHAB, epithelial markers, proteasome-related genes and proliferation markers (Figure 8E,F). These patterns organize epithelial-state heterogeneity and nominate transitions for experimental examination.

### 3.9. Virtual Perturbation, Metabolic Scoring, and CellChat Provide Testable Hypotheses

**Figure 9:**
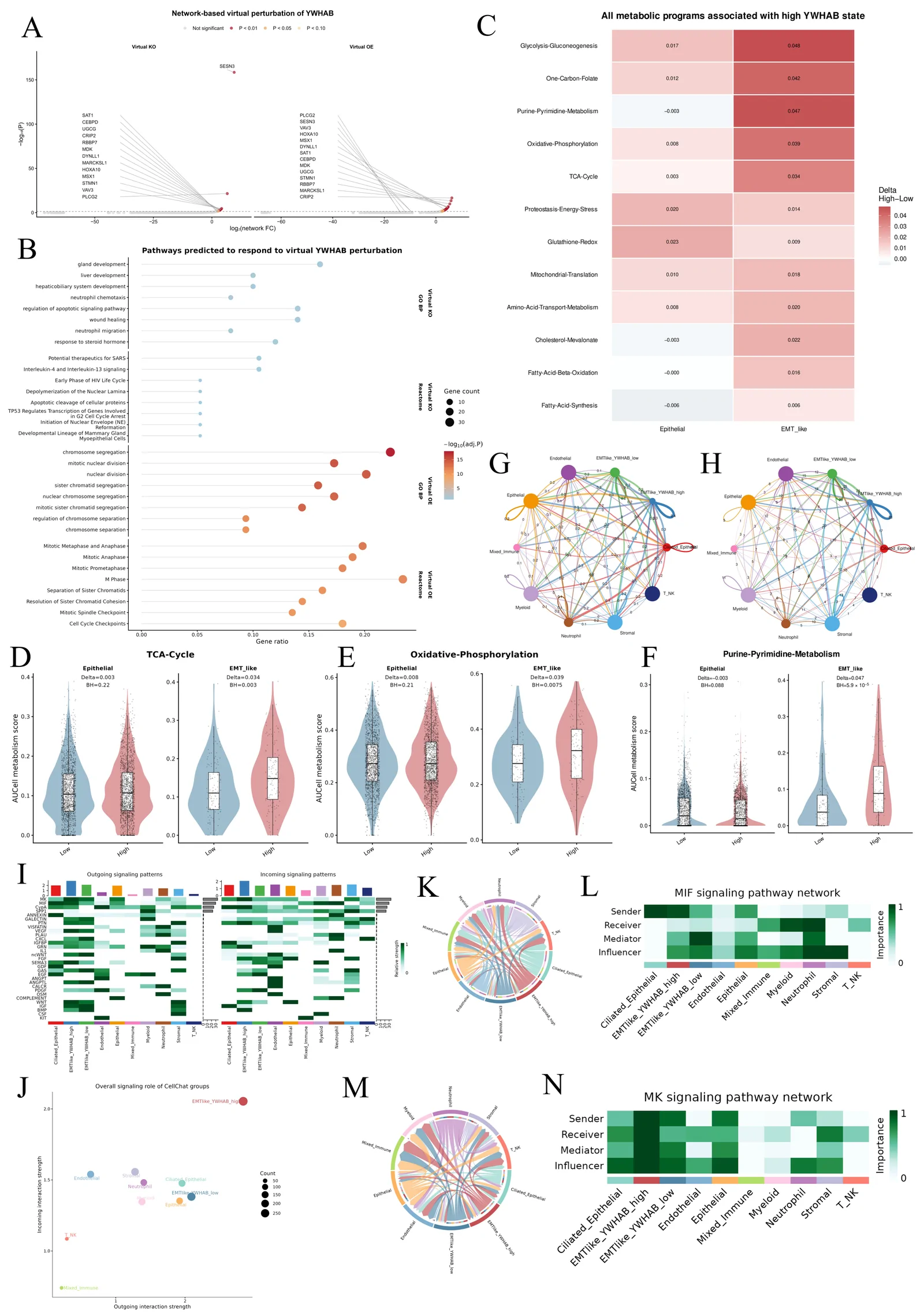
Model-based YWHAB perturbation, metabolic expression programs and communication potential. (**A**) Virtual overexpression response after tenfold amplification of YWHAB network edges. Network fold change is a squared-manifold-distance statistic; colors use raw *p* thresholds and do not represent experimental expression changes. (**B**) Predicted response-gene enrichment. (**C**) High−Low differences across twelve AUCell metabolic expression programs. (**D**–**F**) TCA, oxidative-phosphorylation and purine/pyrimidine score distributions under within-cell-type YWHAB median splits. Epithelial Low/High *n* = 1672/1672 cells; EMT_like *n* = 126/127 cells. Shown *p* values use Wilcoxon tests with BH adjustment across twelve programs per cell type. Scores reflect mRNA programs, not metabolic flux (Supplementary Table S17). Blue and pink violins represent the YWHAB-low and YWHAB-high groups, respectively. (**G**) CellChat communication strength and (**H**) interaction count, followed by pathway role summaries and MIF/MK outputs. Node colors distinguish cell groups, and edge widths represent inferred communication strength and interaction count, respectively. (**I**–**N**) CellChat used the 43,613-cell atlas, downsampled to 17,100 cells, with 523/1293 YWHAB-high/low EMT_like cells. Other cell-type labels were unchanged. Inferred links represent ligand–receptor mRNA communication potential; source outputs are in Supplementary Table S7. Abbreviations: AUCell, area under the recovery curve-based gene-set scoring; TCA, tricarboxylic acid; EMT_like, epithelial-to-mesenchymal transition-like; BH, Benjamini–Hochberg; MIF, macrophage migration inhibitory factor; MK, midkine; mRNA, messenger RNA.

Virtual network perturbation generated predicted responses to amplification of YWHAB-associated network edges (Figure 9A). Response-gene enrichment included gland development, neutrophil chemotaxis and migration, regulation of apoptotic signaling and wound-healing-related processes (Figure 9B; Supplementary Table S7). The response fold-change axis represents a manifold-based network statistic rather than measured gene-expression fold change. These predicted processes provide hypotheses for controlled YWHAB perturbation.

Metabolic-program scores were compared under cell-type-specific YWHAB median splits. Epithelial Low/High groups contained 1672 cells each, and EMT_like groups 126/127 cells. YWHAB-high EMT_like cells had higher oxidative-phosphorylation scores (Δ = 0.03875, BH q = 0.0075), purine/pyrimidine scores (Δ = 0.0468, q < 0.001) and tricarboxylic acid (TCA) scores (Δ = 0.0340, q = 0.00305). Corresponding Epithelial adjusted *p* values were 0.207, 0.088 and 0.220 (Figure 9C–F). Scoring-background sensitivity analyses provided sample-level descriptive summaries: five tumor Epithelial samples were eligible, while only one EMT_like sample met the group-size requirement (Supplementary Table S17). The limited replication constrains interpretation to metabolic transcriptional states rather than established metabolic-flux differences.

CellChat inferred different ligand–receptor mRNA patterns between YWHAB-defined EMT_like groups. The analysis used 17,100 downsampled cells, including 523 EMT_like_YWHAB_high and 1293 EMT_like_YWHAB_low cells. Network and pathway outputs implicated MK, MIF, CypA, SPP1, GRN and CXCL axes (Figure 9G–N; Supplementary Figure S9; Supplementary Table S7). These results describe communication potential within the analyzed atlas rather than tumor-specific protein signaling or YWHAB-mediated regulation.

## 4. Discussion

By integrating single-cell transcriptomics, co-expression networks, genetic associations, patient survival data and tissue protein expression, this study prioritized YWHAB within ubiquitination-related transcriptional programs in EC. Restricting differential expression and co-expression analysis to Epithelial and EMT_like cells reduced the influence of broad cell-lineage composition on candidate selection. Concordance with independently curated UPS gene sets, sample-level sensitivity analyses and cross-model ranking under repeated nested cross-validation provided complementary support for this discovery process. YWHAB was absent from all three scoring gene sets, so its selection did not arise from direct inclusion in the expression signatures. Instead, it reflected associations with the ubiquitination-related transcriptional state and its co-expression network. Functional analyses linked YWHAB-associated states to RNA processing, translation, proteostasis and metabolic adaptation. Together with patient survival associations and tissue protein-expression results, these findings suggest that YWHAB may mark epithelial states with particular biosynthetic and stress-adaptation requirements.

Epithelial plasticity is an important feature of tumor development and progression. EMT is increasingly viewed as a continuum of intermediate phenotypes rather than a simple transition between epithelial and mesenchymal endpoints [20,21]. Cells can retain epithelial characteristics while acquiring programs associated with migration, proliferation, stemness and treatment resistance [22]. Such plasticity involves coordinated changes in adhesion, cytoskeletal organization, metabolism, protein synthesis and stress responses. YWHAB encodes 14-3-3β, a conserved protein that recognizes phosphorylated motifs and influences client-protein stability, subcellular localization and signaling-complex formation [23,24]. Its interactions with cell-cycle, apoptotic and metabolic regulators provide a potential route for connecting these functional networks [23,25]. Studies in several malignancies have linked YWHAB and other 14-3-3 family members to proliferation and invasion [26,27,28]. Gopaul et al. reported that YWHAB knockdown suppressed proliferation, migration and EMT-associated phenotypes in breast cancer cells [29]. These observations motivate examining YWHAB in EC epithelial plasticity, although the EMT_like annotation here describes a transcriptional state without demonstrating an actual EMT.

A biologically relevant finding was the co-enrichment of RNA processing, ribosome biogenesis, protein translation, mitochondrial translation and protein quality-control programs in YWHAB-associated states. These processes coordinate protein production and quality control, thereby contributing to cellular proteostasis. Sustained transcription and translation increase the demand for RNA processing and protein synthesis, alongside folding, complex assembly and clearance of abnormal proteins. The UPS, chaperone networks and related quality-control pathways help balance these demands under changing biosynthetic loads [30,31]. Their coordinated engagement may connect biosynthetic activity with stress adaptation during changes in epithelial state [32,33,34]. YWHAB-associated co-enrichment could reflect this coordination rather than a specific increase in ubiquitination activity. In a neuronal differentiation model, 14-3-3β was implicated in Akt-dependent PRP19α/CDC5L complex formation, linking it to proteins involved in RNA processing [23,35]. Other work showed that 14-3-3 family proteins can direct misfolded, chaperone-associated proteins toward aggresomes [24,36]. These studies provide a molecular context for testing whether 14-3-3β scaffolding connects RNA processing, signaling and proteostasis networks in EC.

Proteostatic adaptation may also be relevant to the maintenance of epithelial plasticity. EMT-associated matrix production, secretion and structural remodeling can increase the endoplasmic-reticulum protein-processing load and engage stress-response pathways. Feng et al. reported sustained PERK–eIF2α signaling during experimental EMT and linked this response to invasion and metastatic capacity [37]. This work suggests a functional connection between endoplasmic-reticulum adaptation and EMT-associated malignant phenotypes. Endoplasmic-reticulum stress has also been studied experimentally in EC tissues and cell models. Bifulco et al. reported increased GRP78, ATF6 and CHOP expression in endometrioid adenocarcinoma and reduced Ishikawa-cell growth after GRP78 knockdown [38]. Together with our translation, folding and quality-control expression patterns, these observations motivate testing whether YWHAB-associated states reflect adaptation to protein-synthesis and folding stress. One hypothesis is that YWHAB-associated protein interactions and stress-response networks participate in maintaining these states. This hypothesis links ubiquitination-related transcription to proteostasis in EC, while leaving YWHAB’s functional contribution to be determined experimentally.

Metabolic analyses extend this proposed connection between biosynthetic demand and epithelial-state adaptation. YWHAB-high EMT_like cells had higher expression scores for oxidative phosphorylation, the tricarboxylic acid cycle and purine/pyrimidine metabolism. The corresponding epithelial comparisons did not reach statistical significance, suggesting a possible context dependence that requires direct testing. The TCA cycle supports mitochondrial energy production while providing metabolic intermediates for biosynthesis, whereas nucleotide metabolism supplies essential precursors for RNA and DNA synthesis. These transcriptional patterns may accompany the energy and precursor demands of RNA processing, translation and cell-state remodeling. Cancer cells can engage glycolytic and oxidative programs according to their state and environment [39,40]. Relationships between EMT, mitochondrial metabolism and survival can vary across tumor contexts [39,41,42]. Experimental breast and liver cancer models further illustrate how EMT can accompany mitochondrial and lipid-metabolic remodeling [43,44]. Muralidharan et al. likewise reported metabolic heterogeneity among epithelial, hybrid and mesenchymal states across cancers [45]. Our observations may describe a particular YWHAB-associated EMT_like state rather than a general metabolic property of EMT. Cell-cycle activity and proliferation-related biosynthetic demand could also contribute to these patterns. Mitochondrial function and metabolic flux measurements would help establish whether the expression differences correspond to altered cellular metabolism.

In the TCGA-UCEC cohort, higher YWHAB expression was significantly associated with poorer OS. Native scSurv also showed positive associations between YWHAB expression and survival-related transcriptional projections in tumor epithelial-lineage cells. Positive correlations persisted after masking direct YWHAB input and refitting the model, indicating that related information was also present among other genes. The broader enrichment patterns suggest that these projected states may involve biosynthetic activity and stress-adaptation requirements. RNA processing and protein synthesis require both metabolic precursors and quality-control systems to sustain intracellular homeostasis. Such coordination could help cells withstand metabolic or microenvironmental pressures and maintain state plasticity. YWHAB’s association with adverse OS may therefore reflect its transcriptional-state context, a possibility that requires functional investigation. MR provided a directionally similar susceptibility signal, but this single-instrument association did not survive multiple-testing correction. Disease susceptibility, patient OS and cellular projections remain distinct endpoints, so their directional concordance supports exploratory prioritization without providing independent validation. These findings motivate testing how YWHAB-associated stress-adaptive states relate to EC progression.

Pseudotime and virtual network perturbation provided further hypotheses about the organization of YWHAB-associated epithelial states. Epithelial and EMT_like cells occupied different regions of the inferred trajectory, with YWHAB expression and related programs varying along the computational ordering. This heterogeneity is consistent with broader single-cell observations of plastic tumor states [46]. It also raises the possibility that state variation accompanies coordinated changes in biosynthetic demand, stress responses and metabolism. The inferred ordering does not establish cellular origin or the direction of an actual transition. Virtual perturbation nominated glandular-development, apoptotic, inflammatory and tissue-repair programs as potential responses within the modeled network. These predictions identify functional questions, including whether experimentally altering YWHAB changes stress responses, apoptosis sensitivity or migration-associated phenotypes.

YWHAB-defined EMT_like states also differed in their predicted cell-communication profiles. CellChat inferred higher components of MK, SPP1 and CypA communication potential in YWHAB-high cells, alongside different patterns for MIF, GRN, CXCL and GALECTIN. The estimates describe ligand–receptor mRNA patterns, rather than measured secretion, receptor activation or signaling flux [47,48]. Midkine has roles in growth-factor signaling, development and repair [49,50], including wound-associated epithelial and inflammatory responses [51]. Osteopontin participates in adhesion, extracellular-matrix organization and inflammation [52], with additional links to regeneration and fibrosis [53,54]. CypA’s associations with protein folding, hypoxia and cellular stress provide a possible connection to the intracellular proteostasis patterns [55,56]. Opposing MIF sender and mediator patterns may reflect altered network organization, consistent with literature on MIF–CD74 epithelial injury and repair responses [57,58]. GRN-related incoming potential and lower CXCL/GALECTIN outgoing potential suggest additional differences in predicted environmental interactions. Literature on progranulin and inflammatory resolution [59,60], chemokine recruitment [61] and galectin-associated tissue remodeling [62] provides context for these hypotheses. These patterns mainly identify communication axes for testing in independently annotated tumor tissues. They do not establish tumor-specific signaling or regulation of these pathways by YWHAB.

Together, these findings support a testable hypothesis that YWHAB marks EC epithelial states with coordinated RNA-processing, protein-synthesis and protein quality-control demands. Some YWHAB-high EMT_like cells additionally show mitochondrial oxidative and nucleotide-metabolism expression programs. These programs may reflect adaptation to state plasticity and environmental pressure, offering a possible biological context for the adverse survival association. Institutional Western blotting and public protein-expression data strengthen the rationale for investigating YWHAB at the tissue level. Loss-of-function, overexpression and rescue experiments should test whether YWHAB contributes to these state-associated programs. Readouts could include ubiquitination load, unfolded-protein-response signaling, mitochondrial function, cell survival and migration. Such experiments would distinguish YWHAB’s potential functional role from its ability to mark a broader adaptive transcriptional state. This study therefore identifies a candidate within ubiquitination-related EC transcription and proposes a biological framework for subsequent experimental evaluation.

## 5. Limitations

Several limitations should be considered when interpreting our findings. First, the single-cell atlas included five tumor and two normal samples from different datasets, with potential source-related confounding and limited biological replication. Although lineage-restricted analyses and paired pseudobulk sensitivity analyses improved interpretability, larger independent cohorts are needed to confirm the observed transcriptional patterns. Second, ubiquitination-related and metabolic scores reflect transcriptional programs rather than direct biochemical activity. The exploratory MR analysis relied on a single whole-blood instrument for YWHAB and did not survive multiple-testing correction. The outcome GWAS and LD reference were European, but the exposure data lacked tissue-specific ancestry information, limiting assessment of ancestry alignment. The 541-patient TCGA-UCEC cohort contained 90 OS deaths. Repeated nested cross-validation strengthened internal assessment, but external prognostic validation and assessment of incremental clinical value remain necessary.

Furthermore, scSurv projections, pseudotime trajectories, virtual perturbation and CellChat analyses provide complementary computational insights into epithelial-state heterogeneity, but do not establish causal regulatory mechanisms. The limited sample-level replication for EMT_like metabolic comparisons also warrants cautious interpretation. Finally, tissue protein-expression results support the relevance of YWHAB in EC but cannot establish its biological function. Future studies incorporating YWHAB perturbation, biochemical and metabolic assays, and independent clinical cohorts will be essential to validate the proposed links among proteostasis, metabolic adaptation and epithelial plasticity.

## 6. Conclusions

Lineage-restricted single-cell discovery, repeated cross-model prioritization and tissue protein-expression evidence identify YWHAB as an exploratory candidate associated with ubiquitination-related transcriptional programs in EC. YWHAB-associated expression patterns connect RNA processing and proteostasis with epithelial-state heterogeneity, accompanied by distinct metabolic signatures in YWHAB-high EMT_like cells and an adverse survival association in TCGA. These findings suggest a potential link between YWHAB-associated transcriptional states and tumor-cell stress adaptation, providing a focused biological framework for functional investigation and future clinical validation.

## Data Availability

The scRNA-seq matrices are available in GEO under GSE173682 and GSE299623; the analyzed sample accessions are listed in Supplementary Table S11. TCGA-UCEC RNA-seq data were obtained from GDC and matched to clinical and OS records. Whole_Blood exposure data came from the Westlake University compilation of GTEx V8 eQTL summaries, and the European-ancestry outcome GWAS was IEU OpenGWAS ebi-a-GCST90018838. Source and instrument records are provided in Supplementary Tables S9 and S14. Curated UPS memberships and MSigDB 2026.1.Hs source records are in Supplementary Table S13. Public immunohistochemical images are available from the Human Protein Atlas. Analysis code and key parameter files are available from the corresponding author upon reasonable request. Result tables and the complete institutional Western blot measurements and uncropped images are included in the Supplementary Material.

## Supplementary Materials

The following supporting information can be downloaded at: https://www.mdpi.com/article/10.3390/genes17101316/s1, Figure S1: Marker expression in the integrated single-cell atlas; Figure S2: Three-dimensional PCA visualization of the single-cell atlas; Figure S3: Inferred cell-cycle phases in the single-cell atlas; Figure S4: Mean expression of ubiquitination-related genes by cell type and tissue group; Figure S5: Distributions of individual ubiquitination-related expression scores across the atlas; Figure S6: hdWGCNA soft-thresholding diagnostics; Figure S7: Module-membership rankings in the retained hdWGCNA network; Figure S8: Disease Ontology enrichment of the revised 444 candidate genes; Figure S9: CellChat-inferred ligand–receptor communication involving YWHAB-defined EMT_like groups; Figure S10: Concordance of five individual scores and the consensus score in the revised epithelial lineage; Figure S11: Kaplan–Meier curves for YWHAB expression in a fixed training and outer-test split; Figure S12: Cell-level and paired sample-level Q4 versus Q1 differential-expression sensitivity; Figure S13: Sensitivity analysis using independently curated UPS gene sets; Figure S14: Native scSurv input-masking and random-seed sensitivity; Figure S15: Western blot images and HPA immunohistochemical staining of YWHAB; Table S1: Top 20 markers for each of 24 original atlas clusters; Table S2: GeneCards-derived ubiquitination gene set and detected members; Table S3: Complete cell-level epithelial-lineage Q4 versus Q1 differential expression; Table S4: High-score-associated hdWGCNA core genes and module selection; Table S5: 444 intersection candidates, TCGA mapping and functional enrichment; Table S6: Exploratory MR and cross-model candidate prioritization; Table S7: YWHAB expression associations, original YWHAB-expression enrichment, virtual perturbation and CellChat; Table S8: Five-method and consensus score concordance in the epithelial lineage; Table S9: MR data sources, selection rules and instrumental variants; Table S10: YWHAB continuous-expression Cox analysis and fixed-cutoff Kaplan-Meier groups; Table S11: Sample accession, histology, tissue and frozen atlas cell counts; Table S12: Paired sample-aware pseudobulk and candidate-set sensitivity; Table S13: Independently curated UPS memberships and UCell concordance sensitivity; Table S14: Complete MR candidate flow, primary results and harmonization; Table S15: Updated TCGA cohort and repeated nested cross-validation; Table S16: Native scSurv 32,263 full/masked paired seed sensitivity; Table S17: Original metabolic program comparisons and cell/sample sensitivity analyses; Table S18: YWHAB Western blot quantification and original image index.

## Abbreviations

The following abbreviations are used: AUCell, area under the recovery curve-based gene-set scoring; BP, biological process; CC, cellular component; C-index, concordance index; DEG, differentially expressed gene; DO, Disease Ontology; EC, endometrial carcinoma; EMT, epithelial-to-mesenchymal transition; EMT_like, epithelial-to-mesenchymal transition-like; FDR, false discovery rate; FPKM-UQ, fragments per kilobase per million with upper-quartile normalization; GDC, Genomic Data Commons; GEO, Gene Expression Omnibus; GO, Gene Ontology; GSEA, gene-set enrichment analysis; GWAS, genome-wide association study; hdWGCNA, high-dimensional weighted gene co-expression network analysis; HPA, Human Protein Atlas; IEU, MRC Integrative Epidemiology Unit; KEGG, Kyoto Encyclopedia of Genes and Genomes; LD, linkage disequilibrium; MF, molecular function; MIF, macrophage migration inhibitory factor; MK, midkine; MR, Mendelian randomization; OS, overall survival; PCA, principal component analysis; QL, quasi-likelihood; scRNA-seq, single-cell RNA sequencing; ssGSEA, single-sample gene-set enrichment analysis; TCA, tricarboxylic acid; TCGA-UCEC, The Cancer Genome Atlas Uterine Corpus Endometrial Carcinoma; UMAP, Uniform Manifold Approximation and Projection; UPS, ubiquitin–proteasome system; YWHAB, tyrosine 3-monooxygenase/tryptophan 5-monooxygenase activation protein beta.
